# Hypofractionated stereotactic radiation therapy activates the peripheral immune response in operable stage I non-small-cell lung cancer

**DOI:** 10.1038/s41598-017-04978-x

**Published:** 2017-07-07

**Authors:** Ting Zhang, Haifeng Yu, Chao Ni, Tao Zhang, Luying Liu, Qinghua Lv, Zhigang Zhang, Zhen Wang, Dang Wu, Pin Wu, Guodi Chen, Liancong Wang, Qichun Wei, Jian Huang, Xiaojian Wang

**Affiliations:** 1 Department of Radiation Oncology, The Second Affiliated Hospital, Zhejiang University School of Medicine, Zhejiang University, Hangzhou, 310009 China; 2 Department of Chemotherapy Center, Zhejiang Cancer Hospital, Hangzhou, 310022 China; 3 Department of General surgery, Zhejiang Provincial People’s Hospital, Hangzhou, 310014 China; 4 Cancer Institute (Key Laboratory of Cancer Prevention & Intervention, National Ministry of Education; Provincial Key Laboratory of Molecular Biology in Medical Sciences), Second Affiliated Hospital, Zhejiang University School of Medicine, Zhejiang University, Hangzhou, 310009 China; 5 Institute of Immunology, School of Medicine, Zhejiang University, Hangzhou, 310058 China

## Abstract

It has been reported that in patients with operable stage I non-small cell lung cancer (NSCLC), overall survival (OS) is better in those who undergo hypofractionated stereotactic radiation therapy (HSRT) than in those who undergo surgery. However, the reason that HSRT has a better OS has not been fully explored. Here, we analyzed reconstitution kinetics in immune cells in the peripheral blood of NSCLC patients after HSRT. We found that HSRT increased the frequency of total T cells, especially the proportion of CD8^+^ T cells, but decreased the frequency of inhibitory Tregs. Intracellular staining showed that after HSRT, peripheral CD8^+^ T cells were transformed into activated T cells, which express high levels of TNF-α, IFN-γ, granzyme B and IL-2. HSRT also increased the production of IL-2, TNF-α, and IFN-γ but down-regulated the production of TGF-β in CD4^+^ T cells. The frequencies of naïve B cells and double-negative B cells were lower, while the proportions of MZ-like B cells, transitional B cells and plasmablast cells were higher after HSRT. Collectively, our results demonstrate that HSRT activates the peripheral immune response and indicate the dynamic variation in peripheral lymphocytes after HSRT, which is very important for optimizing combination treatments in clinical practice.

## Introduction

Approximately 60% of patients with solid tumors, including newly diagnosed cancers and persistent or recurrent tumors, receive radiotherapy (RT) with the explicit goal of eliminating tumors through direct killing^1, 2^.

Hypofractionated stereotactic radiation therapy (HSRT) is a modern radiation technique that provides precisely targeted high-dose irradiation to a tumor while limited damage to surrounding normal tissues^3^. Recently, Chang demonstrated that in patients with operable stage I non-small cell lung cancer (NSCLC), overall survival (OS) was much better in an HSRT group than a surgery group^4^. However, the reason for the prolonged OS conferred by HSRT has not been determined.

In general, surgery induces a transient depression in lymphocyte functions in the peripheral blood of cancer patients^5^, whereas RT enhances immune responses in both the tumor microenvironment and the immune system. RT can also induce immunogenic cancer cell stress or death and promote the transfer of calreticulin to cancer cell plasma membranes and the release of ATP and HMGB1. These factors bind to CD91, P2RX7, and TLR4, which are expressed on dendritic cells (DCs), to recruit DCs into the tumor bed. Once there, the DCs engulf tumor antigens and present them to T cells^6–9^. RT also reprograms tumor macrophages to become M1 cells^10^ and induces the secretion of chemokines, such as CXCL16^11^, which enable T cells to home to the tumor site, where they can activate the immune response. Interestingly, clinical studies have revealed that RT can provoke tumor cell responses not only at the site of treatment but also in remote, non-irradiated tumor deposits via what is called an “abscopal effect”^12, 13^. Collectively, these studies indicate that surgery and radiation affect the immune response differently.

In 1953, Mole coined the term “abscopal” to describe the systemic effect of radiation on “out-of-field” tumor deposits^14^. Since then, the abscopal effect has been reported in many types of tumors that are treated with HSRT^15–17^, and it is more commonly observed when HSRT is combined with immunotherapy^13, 18, 19^. The abscopal effect was observed in up to 27% of patients with metastatic solid tumors who were treated with concurrent HSRT at one metastatic site in combination with a GM-CSF subcutaneous injection^20^. Combining radiotherapy and immunotherapy may be the next step in oncology practice^18^. However, this approach has not yet been fully explored as a therapy, and when and how HSRT should be combined with immunotherapy to achieve a maximum effect and how the effects of this treatment should be evaluated remain unknown. Studies that explore these points will be important for implementing individualized treatment. Determining *in vivo* peripheral immune responses at different times after HSRT may be helpful in designing the best regimen for this combined treatment.

Many studies have used immunohistochemistry assays to examine subsets of immune cells in tumor sites in tissues obtained from patients treated with HSRT. These reports have demonstrated that CD8^+^ cytotoxic lymphocytes (CTLs) and CD4^+^ T cells are indispensable for the therapeutic effects of HSRT^21^. The function of B cells in the tumor microenvironment is controversial^22, 23^. Different B cell subsets play different roles in anti-cancer immunity. However, the dynamics of the changes that occur in peripheral immune cell compositions post-HSRT are poorly identified.

In this study, we first describe the dynamics of the changes that occur in the peripheral immune response post-HSRT. We enrolled 6 patients with operable lung cancer who underwent HSRT, and we determined the proportions of subsets of immune cells, including T cells, B cells, NK cells, and Tregs, and the levels of cytokines produced in the PB obtained from these patients at different timepoints after HSRT.

## Materials and Methods

### Clinical patients and study design

For this study, we designed a strategy to examine variations in lymphocyte subsets in stage I NSCLC patients who were treated with HSRT from August 2010 until now. After they signed an informed consent document, 6 NSCLC patients who did not undergo surgery for tumor removal but were treated with HSRT were enrolled in this study. All of the patients were negative for antibodies against the hepatitis C virus and hepatitis B virus, HIV, and syphilis. Approximately 10 mL of heparinized peripheral blood was collected from each patient at the selected times. The protocol was approved by the ethics board of The Second Affiliated Hospital of Zhejiang University. The demographic and clinical data for the patients are summarized in Table 1. All of the patients experienced tolerable side effects and displayed no progression during the follow-up period (Table S1).

**Table 1:** Patient demographics.

	HSRT(n = 6)
Sex	
Male	4(67%)
Female	2(33%)
Age(years)	
Mean(SD)	76.7
Median(range)	77
WHO performance status	
0	3(50%)
1	3(50%)
2	0
Histology before therapy	
Adenocarcinoma	3(50%)
Squamous	1(17%)
Unknown	2(33%)
Tumor stage	
T1a	3(50%)
T1b	1(17%)
T2a	2(33%)
Stage	
IA	4(67%)
IB	2(33%)
Tumor site	
Left lower lobe	1(17%)
Left upper lobe	3(50%)
Right lower lobe	0
Right middle lobe	0
Right upper lobe	2(33%)

Data are n (%) unless otherwise stated. HSRT, hypofractionated stereotactic radiotherapy.

### HSRT therapy

A moderate dose of HSRT was used because a higher dose of RT is not clearly correlated with a better immune response but is likely to increase toxicity. Patients were treated with HSRT using the following dose regimen: 48 Gy/8 F or 48 Gy/6 F according to their pulmonary function. A total dose of 48 Gy was delivered in 6 or 8 fractions using a 6-MV X-ray. Plan normalized at 100% prescription cover 95% target volume. RT was performed using a three-dimensional treatment plan across several different non-coplanar fixed fields in a linear accelerator (Varian). No patients were treated with chemotherapy or steroids.

### Blood Sampling and Flow cytometry

PB samples were collected the day before HSRT was initiated (pretreatment, pre), immediately after the completion of HSRT (post-treatment, pos) and at 3 weeks post-treatment (pos-3w). The peripheral blood specimens were layered over Ficoll-Paque (GE Healthcare) and then centrifuged for 30 min at 1,500 rpm. The interface containing mononuclear cells was collected and washed in PBS twice. The mononuclear cells were stained for flow cytometric analysis using a fluorescence-labeled monoclonal antibody^24^.

### Flow cytometry

For extracellular staining, the cells were first pre-incubated in a mixture of PBS, 2% fetal calf serum, and 0.1% (w/v) sodium azide that contained an FcγIII/IIR-specific antibody to block nonspecific binding. The sections were then stained with different combinations of fluorochrome-coupled antibodies (Table S2). For intracellular staining, the cells were activated using Leukocyte Activation Cocktail (BD PharMingen) for 6 h according to the manufacturer’s protocol. The cells were then collected using a FACSCanto II system (BD Biosciences), and the data were analyzed using FlowJo software (Tree Star).

### Statistical analysis

Statistical analyses were carried out using the IBM SPSS Statistics software (v.24.0.0.0, International Business Machines, Armonk, NY). Data are expressed as means ± SEM. Statistics were derived using ANOVA with Bonferroni’s post-test. P value < 0.0167 was considered to indicate statistically significant. *p < 0.0167, **p < 0.01; ***p < 0.001. Prism Graph Pad version 5 was used for all statistical calculations.

### Ethical Approval and Informed Consent

This study was approved by the Ethical Committee of the Second Affiliated Hospital, Zhejiang University. The patients were interviewed prior to inclusion in the study and were provided with written and verbal information regarding the study. All patients provided written informed consent. All methods were performed in accordance with the relevant guidelines and regulations.

## Results

### Dynamic peripheral lymphocyte composition after HSRT

**Figure 1:**
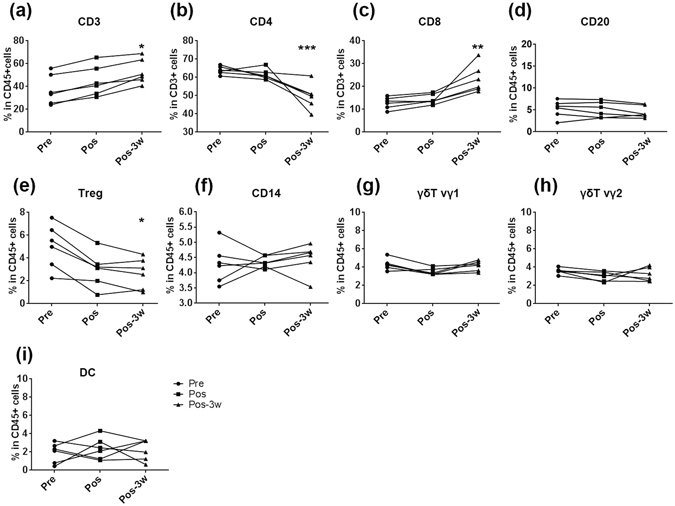
Peripheral lymphocyte compositions are dynamic after HSRT. (**a**) Peripheral blood samples were obtained from the 6 enrolled NSCLC patients on the day before HSRT (pretreatment, pre; dark circle), immediately after the completion of HSRT (post-treatment, pos; dark square) and at 3 weeks post-treatment (pos-3w; dark triangle). PBMCs were harvested and labeled with fluorochrome-coupled antibodies. The percentage of CD3^+^ T cells in CD45^+^ cells (**a**), CD4^+^ T cells in CD3^+^ T cells (**b**), CD8^+^ T cells in CD3^+^ T cells (**c**), CD20^+^ B cells in CD45^+^ cells (**d**), CD4^+^Foxp3^+^ cells in CD45^+^ cells (**e**), CD14^+^ cells in CD45^+^ cells (**f**), TCRvδ1^+^ cells in CD45^+^ cells (**g**), TCRvδ2^+^ cells in CD45^+^ cells (**h**), and DC in CD45^+^ cells (**i**) were analyzed. All statistical analyses were performed versus Pre(pretreatment) using ANOVA with Bonferroni’s post-test. *p < 0.0167, **p < 0.01; ***p < 0.001.

A previous study reported that in patients with clinical T1-2a (≤4 cm) N0M0 (UICC), operable NSCLC, the estimated OS at 3 years was 95% in a stereotactic RT group and 79% in a surgery group^4^. The composition and function of circulating lymphocytes in patients who underwent HSRT has not yet been described. Here, 6 operable NSCLC patients were enrolled in this study and executed with HSRT. All of these patients were followed up and no disease progression were observed (Fig. S1). We first analyzed variation in the proportions of subsets of lymphocyte in the PB of stage I lung cancer patients who were treated with HSRT. The frequency of CD3^+^ T lymphocytes was slightly higher after HSRT and was significantly higher at 3 weeks after HSRT (P = 0.0161, Fig. 1a). The proportion of CD4^+^ T cells in CD3^+^ T was lower (P = 0.0007, Fig. 1b), while the CD8^+^T/CD3^+^T ratio was markedly higher at 3 weeks post-HSRT (P = 0.0027, Fig. 1c). The percentage of B cells remained nearly stable in HSRT-treated patients, compared with that of the untreated self-controls (Fig. 1d).

Treg cells play important roles in maintaining peripheral tolerance, limiting inflammation, and preventing autoimmunity by inhibiting T-effector cells, which leads to tumor immune tolerance^25^. As shown in Fig. 1e, the frequency of CD4^+^Foxp3^+^ Tregs was lower at 3 weeks post-HSRT (P = 0.0143). Additionally, we examined the other cell subsets, including CD14^+^ monocytes (Fig. 1f), vδ1 γδ T cells (Fig. 1g), vδ2 γδ T cells (Fig. 1h), CD3^−^CD56^+^ NK cells (data not shown), BDCA2^+^pDC cells (data not shown) and CD11c^+^cDC cells (Fig. 1i) and found that the frequencies of these cells were not changed after HSRT. Collectively, these results show that HSRT increased the frequency of the total T cells and the proportion that were CD8^+^ T cells but reduced the frequency of inhibitory Tregs, indicating that HSRT activated the peripheral immune response.

### HSRT induces peripheral CD8^+^ T cell activation

**Figure 2:**
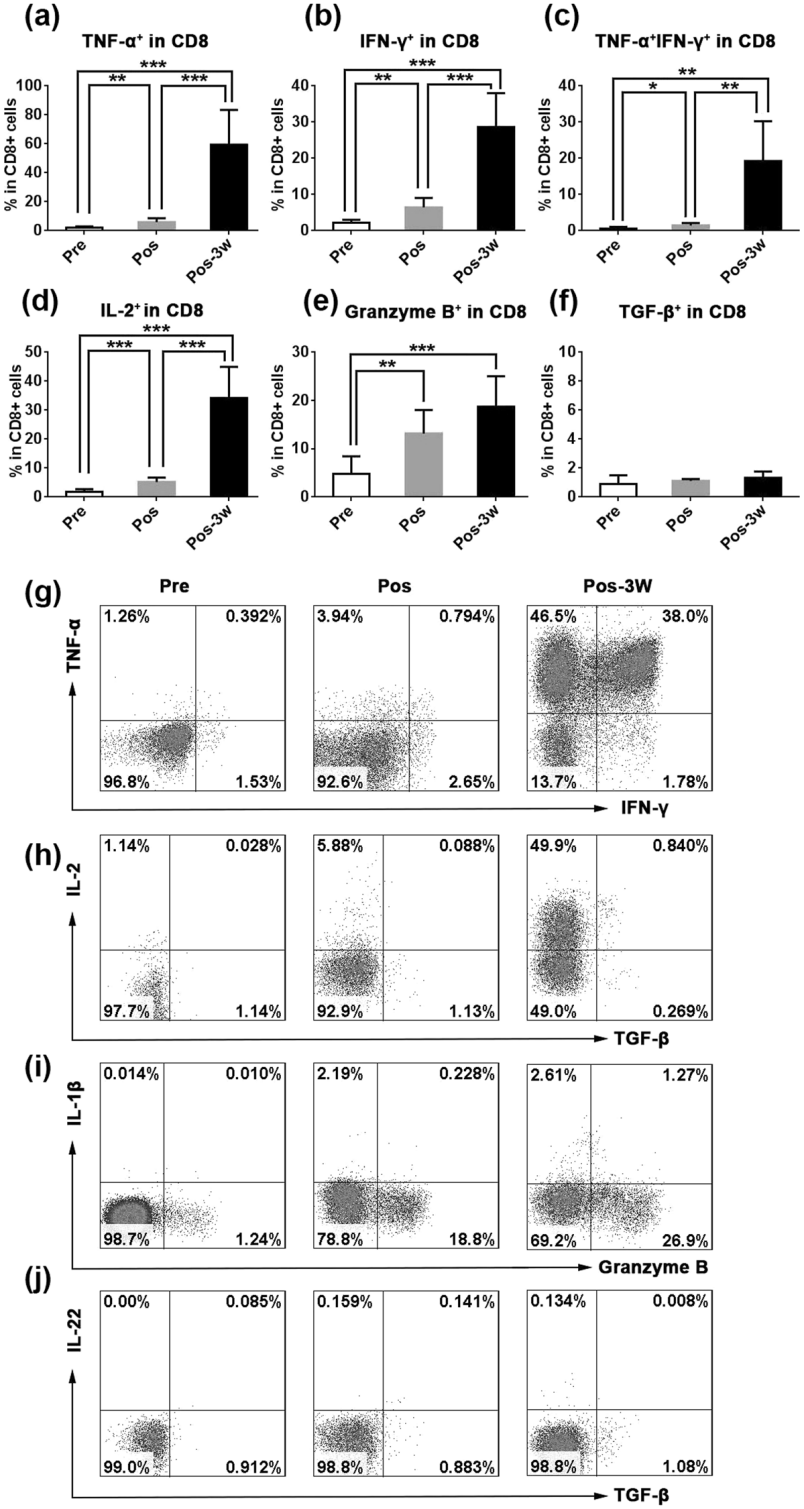
Expression profiling of cytokines expressed by CD8^+^ T cells in peripheral blood after HSRT. PBMCs were treated with a leukocyte activation cocktail for 6 h, and the cells were then collected and labeled with IFN-γ (**g**), TNF-α (**g**), IL-2 (**h**), TGF-β (**h**), IL-1β **(i**), granzyme B (**i**), or IL-22 (**j**). The mean values of the frequencies of TNFα^+^CD8^+^ T cells in CD8^+^ T cells (**a**), IFNγ^+^CD8^+^ T cells in CD8^+^ T cells (**b**), TNFα^+^IFNγ^+^CD8^+^ T cells in CD8^+^ T cells (**c**), IL-2^+^CD8^+^ T cells in CD8^+^ T cells (**d**), granzyme B^+^ CD8^+^ T cells in CD8^+^ T cells (**e**), and TGFβ^+^CD8^+^ T cells in CD8^+^ T cells (**f**) were analyzed at the indicated times. The panel g–j showed the representative result from one patient. The data are shown as the mean values ± SEM (n = 6). Statistics were derived using ANOVA with Bonferroni’s post-test. *p < 0.0167, **p < 0.01; ***p < 0.001.

CTLs are the most important anticancer effective cells^26^. When combined with the delivery of antigenic peptides to the tumor, RT is sufficient to induce the priming and expansion of antigen-specific CTLs, which led to synergistic therapeutic antitumor effects in mice^27^. In humans, the high rate at which CD3^+^ and CD8^+^ lymphocytes infiltrate tumor tissues (as observed in biopsies) was associated with down-staging in rectal cancer following preoperative chemoradiation therapy^28^. Activated T cells express pleiotropic cytokines, including TNF-α, IFN-γ and IL-2. We used intracellular staining for TNF-α and IFN-γ in combination with a panel of surface antibodies to study peripheral blood mononuclear cells (PBMCs) obtained before and after HSRT in 6 patients. TNF-α^+^CD8^+^ T cells were rarely detected in PB before HSRT, but at 3 weeks post-HSRT, they were present in markedly higher numbers (17-fold higher) in treated patients than in untreated self-controls (P = 0.0002, Fig. 2a). The frequency of IFN-γ^+^CD8^+^ T cells increased the day after HSRT (P = 0.0036) and 14-fold higher 3 weeks later (P < 0.0001) than was observed in PB samples obtained before HSRT (Fig. 2b). The frequency of TNF-α^+^ IFN-γ^+^CD8^+^ T was 35-fold higher 3 weeks later (P = 0.0019) than was observed in PB samples obtained before HSRT (Fig. 2c). And the frequency of TNF-α^+^CD8^+^ T cells and IFN-γ^+^CD8^+^ T cells almost restored to pre-RT levels at 3–5 months after HSRT (Fig. S2a,b). Additionally, as shown in Fig. 2d, the frequency of IL-2^+^CD8^+^ T cells was 3-fold higher the day after HSRT (P = 0.0007) and 16-fold higher at 3 weeks after HSRT (P < 0.0001) than was observed in the untreated self-controls. Granzyme B is a serine protease that is released by cytoplasmic granules within CTLs and NK cells that causes target cell death^29^. Here, we found that the frequency of granzyme B^+^ CD8^+^ T cells was higher after HSRT (P = 0.007) and had increased to an even higher level at 3 weeks after HSRT (P = 0.0009, Fig. 2e) than was observed in PB obtained from patients without HSRT. TGF-β inhibits T cell-mediated tumor clearance *in vivo* by specifically inhibiting the expression of five cytolytic gene products, including perforin, granzyme A, granzyme B, Fas ligand, and IFN-γ, in CTLs^30^. However, there was no difference in the level of TGF-β that was produced by CD8^+^ T cells before and after HSRT (Fig. 2f). Taken together, these results suggest that HSRT induces the activation of peripheral CD8^+^ T cells.

### HSRT induces CD4^+^ T cells to reprogram to become more functional T cells in peripheral blood

**Figure 3:**
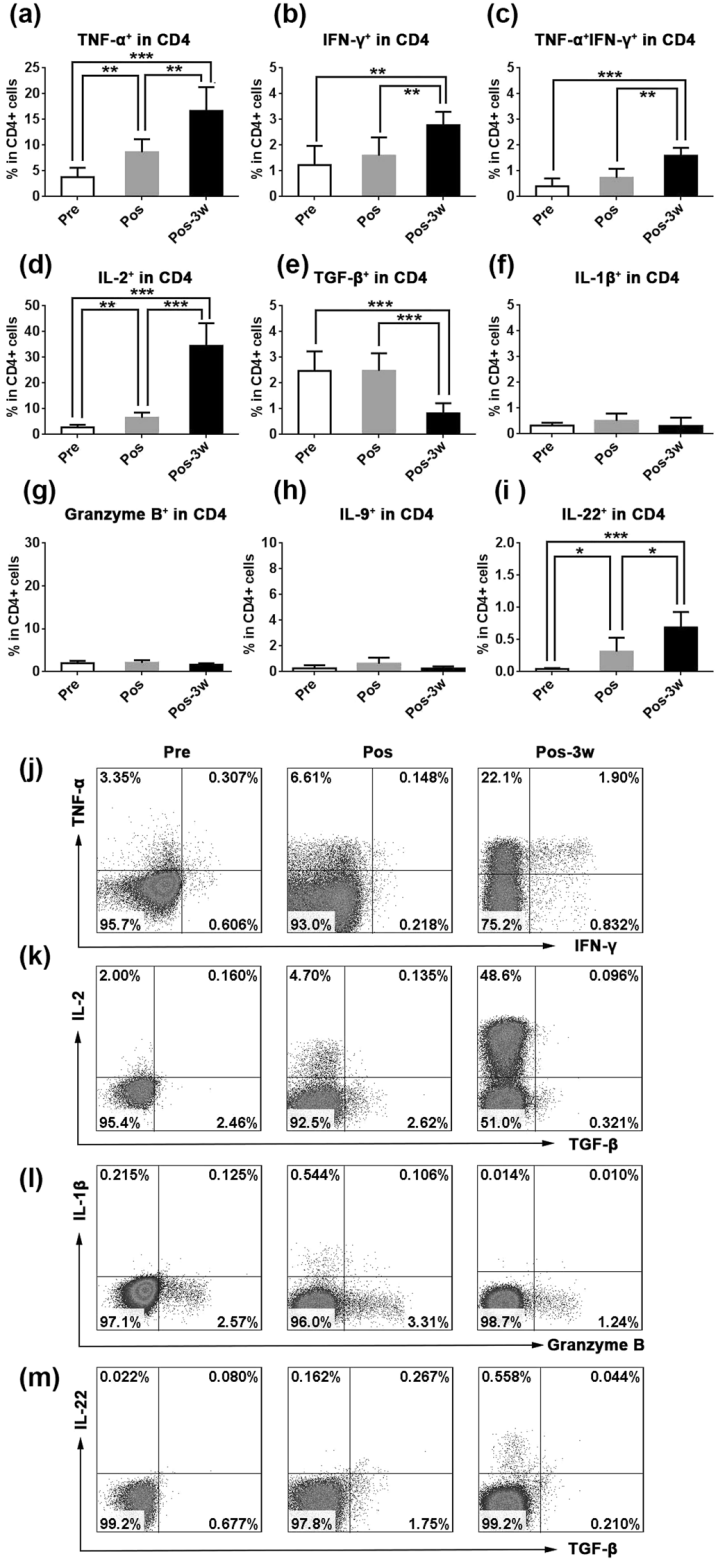
Cytokine secretion by peripheral CD4^+^ T cells after treatment with HSRT. PBMCs were activated using a leukocyte activation cocktail for 6 h, and the cells were then collected and labeled with IFN-γ (**j**), TNF-α (**j**), IL-2 (k), TGF-β (**k**), IL-1β (**l**), granzyme B (**l**), or IL-22 (**m**). The mean values of the percentage of cells that were TNFα^+^CD4^+^ T cells in CD4^+^ T cells (**a**), IFNγ^+^CD4^+^ T cells in CD4^+^ T cells (**b**), TNFα^+^IFNγ^+^CD4^+^ T cells in CD4^+^ T cells (**c**), IL-2^+^CD4^+^ T cells in CD4^+^ T cells (**d**), TGF-β^+^CD4^+^ T cells in CD4^+^ T cells (**e**), IL-1β^+^CD4^+^ T cells in CD4^+^ T cells (**f**), granzyme B^+^ CD4^+^ T cells in CD4^+^ T cells (**g**), IL-9^+^CD4^+^ T cells in CD4^+^ T cells (**h**), and IL-22^+^CD4^+^ T cells in CD4^+^ T cells (**i**) were analyzed at the indicated times. The panel j–m showed the representative result from one patient. The data are shown as the mean values ± SEM (n = 6). Statistics were derived using ANOVA with Bonferroni’s post-test. *p < 0.0167, **p < 0.01; ***p < 0.001.

As T helper cells, CD4^+^ T cells play an important role in anti-cancer immunity. The loss of intrahepatic CD4^+^ T lymphocytes leads to accelerated hepatocarcinogenesis^21^. In some cancers, including melanoma and epithelial cancer, the effect of immunotherapy is based on specific CD4^+^ T cell-mediated cancer regression^31, 32^. Here, we analyzed the production of cytokines, including IL-2, TNF-α, and IFN-γ, in CD4^+^ T cells obtained from peripheral blood after HSRT. As shown in Fig. 3a, the frequency of TNF-α^+^CD4^+^ T cells was 2-fold higher the day after HSRT (P = 0.0035) and 4-fold higher 3 weeks (P < 0.0001) than was observed in untreated self-controls. The proportion of IFN-γ^+^CD4^+^ T cells was approximately 2-fold higher at 3 weeks post-HSRT than was observed in untreated self-controls (P = 0.0019, Fig. 3b). The percentage of TNF-α^+^ IFN-γ^+^CD4^+^ T was also higher at 3 weeks post-HSRT (P = 0.0019, Fig. 3c). IL-2 is crucial for helper T cell activation and expansion. As shown in Fig. 3d, CD4^+^ T cells expressed a high level of IL-2 on the day after HSRT (P = 0.0021), and the number of these cells was 20-fold higher after HSRT than was observed in untreated self-controls (P < 0.0001). Importantly, the level of the inhibitory cytokine TGF-β, which is expressed by CD4^+^ T cells, was decreased at 3 weeks after HSRT (P = 0.0009, Fig. 3e). The frequency of IL-2^+^CD4^+^ T cells and TGF-β^+^CD8^+^ T cells almost restored to pre-RT levels at 3-5 months after HSRT (Fig. S2c,d). The levels of other cytokines, including IL-9, IL-1β, and granzyme B, that are also expressed by CD4^+^ T cells were not significantly altered after HSRT (Fig. 3f–h). IL-22 plays an important role during tissue regeneration by acting on nonhematopoietic epithelial and stromal cells to induce their proliferation^33^. As shown in Fig. 3i, the frequency of IL-22^+^CD4^+^ T cells was increased after HSRT (P = 0.0113). Taken together, these results suggest that in NSCLC patients, HSRT induces CD4^+^ T cells in peripheral blood to be reprogrammed into more functional T cells.

### HSRT regulates the reconstitution of B cell subsets in peripheral blood

**Figure 4:**
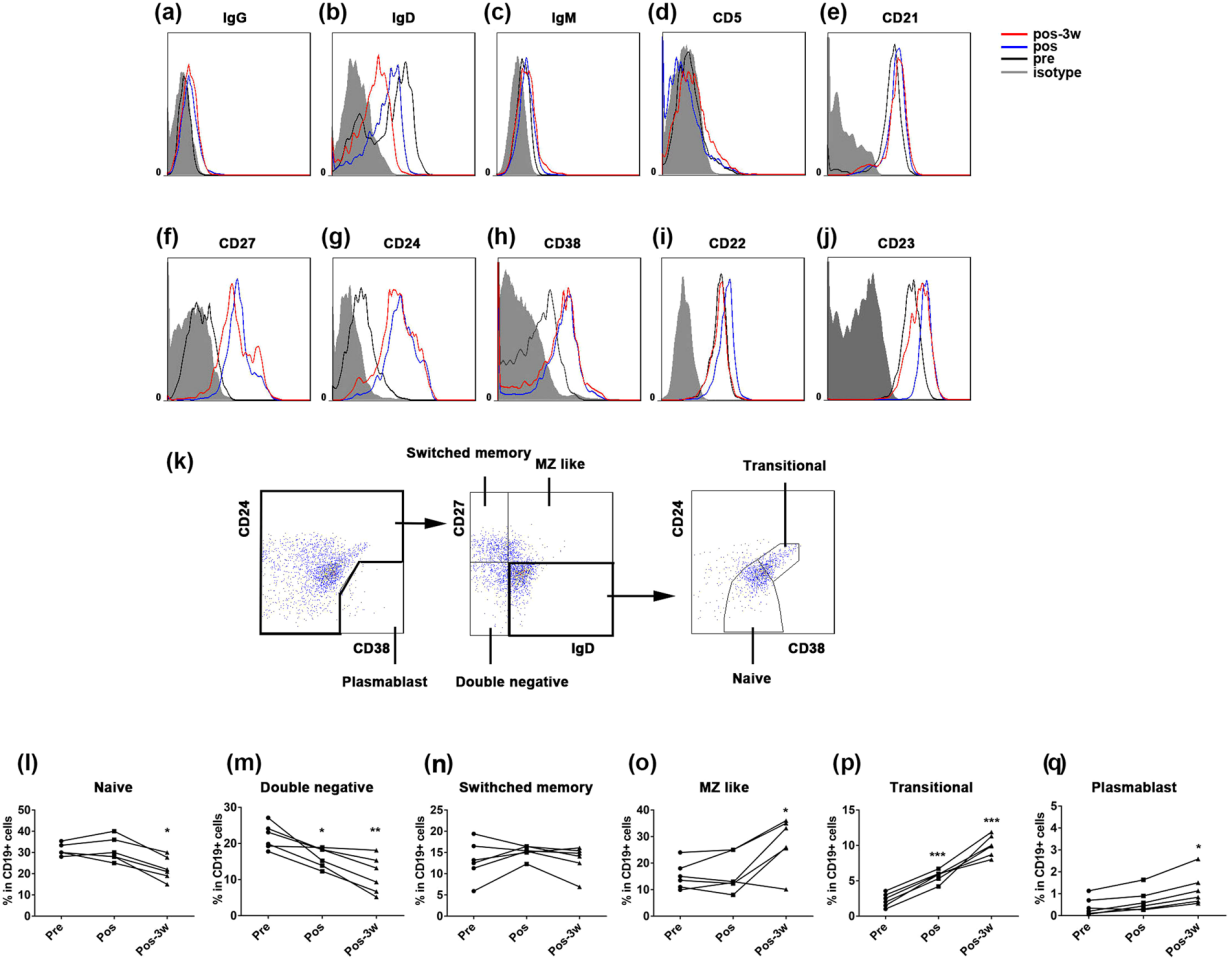
Phenotypes and subpopulation analysis of peripheral B cells after HSRT. (**a**–**j**) The phenotypes of peripheral CD20^+^ B cells are shown before and after treatment with HSRT. Filled grey indicates the isotype, unfilled black indicates pretreatment, unfilled blue indicates post-treatment, and unfilled red indicates 3 weeks post-treatment. (**k**) The gating strategy that was used to identify human B-cell subsets. (**l**–**p**) The percentages of cells that were naïve B cells (**l**), double-negative B cells (**m**), switched-memory B cells (**n**), MZ-like B cells (**o**), transitional B cells (**p**), and plasmablasts (**q**) in CD20^+^ B cells were analyzed at the indicated timepoints before and after HSRT. Dark circles indicate pretreatment, dark squares indicate post-treatment, and dark triangles indicate 3 weeks post-treatment. All statistical analyses were performed versus Pre(pretreatment) using ANOVA with Bonferroni’s post-test. *p < 0.0167, **p < 0.01; ***p < 0.001.

B lymphocytes act not only as antigen presentation cells but also as important antibody-producing cells. Little is known about the post-RT reconstitution kinetics of human peripheral blood CD20^+^ B cell subsets. We investigated the phenotypes of B cells after HSRT and, as shown in Fig. 1d, the percentage of CD20^+^ B cells in CD45^+^ lymphocytes was not significantly different after HSRT. Additionally, Fig. 4a–j shows that B cells exhibit higher expression levels of CD27, CD24, CD38, CD22, CD23, and IgG and lower expression levels of IgD. The expression level of IgM was constant between pre- and post-HSRT (Fig. 4c). These results demonstrate that post-HSRT, B cells display an activated phenotype. We next analyzed subsets of CD19^+^ B cells. We used flow cytometry to gate B cell subsets, as shown in Fig. 4k ^34^. The percentages of cells that were virgin, naïve B cells (defined as IgD^+^CD27^−^CD38^−^) and double-negative B cells (IgD^−^CD27^−^) were decreased after HSRT (Fig. 4l,m), whereas the proportion of switched memory B cells (IgD^−^CD27^+^) in the peripheral blood was not different between post-HSRT samples (Fig. 4n) and untreated self-controls. The percentage of MZ-like B cells was clearly increased at 3 weeks after HSRT (p = 0.0109, Fig. 4o). Moreover, the frequency of transitional B cells was markedly higher the day after HSRT (p < 0.0001), and approximately 10% of the total B cells were transitional B cells at 3 weeks post-HSRT (p < 0.0001, Fig. 4p). HSRT also increased the frequency of plasmablast cells (p = 0.0051, Fig. 4q). Collectively, these results indicate that HSRT regulates the composition of B cell subsets in the peripheral blood of NSCLC patients.

## Discussion

All 6 of the patients who enrolled in this study were followed up and suffered no obvious side effects or recurrence or metastasis. Chang reported that surgery and HSRT were equally effective in treating tumors. The lower survival rates after surgery were potentially the result of surgical complications^4^. Here, we show that HSRT increased the frequency of total T cells, especially the proportion of CD8^+^ T cells, but down-regulated the proportion of inhibitory Tregs. Moreover, after HSRT, peripheral CD8^+^ T cells transformed into activated CTLs, which express high levels of TNF-α, IFN-γ, granzyme B and IL-2. HSRT also increased the production of IL-2, TNF-α, and IFN-γ but decreased the production of TGF-β in CD4^+^ T cells.

It has been reported that radiation therapy has immunosuppressive effect. Immune cells, especially lymphocytes, are very sensitive to radiation. In the rat model of cervical cancer, the up-regulation of programmed cell death-1(PD-1) and programmed cell death-Ligand 1(PD-L1) was observed in cervical cancer tissues at 1 month after RT. In addition, the percentages of CD4^+^T cells and CD8^+^T cells were significantly decreased in the RT group^35^. H. van Meir reported that external beam radiation therapy (EBRT) treatment decreased the frequency of both CD4^+^ and CD8^+^ T cells and enhances the PD-1 expression in CD4^+^ T cells displayed in the patients with cervical cancer^36^. Dewan *et al*. reported that fractionated doses, but not single doses, induced immune-mediated abscopal effects when administered in combination with a CTLA-4 antibody treatment^37^. In mice, Schaue *et al*. found that single doses of radiation ranging from 7.5 to 15 Gy had a dose-dependent impact on tumor control. However, this effect was offset at the highest dose, 15 Gy, by an increase in the number of Treg cells^38^. A fractionated treatment consisting of a medium-sized dose of radiation, 7.5 Gy/fraction, provided the best tumor control and anti-tumor immunity effects while still maintaining low numbers of Tregs^34^. Our results are the first to show that in humans, HSRT with a regimen of 48 Gy/6 F or 48 Gy/8 F induced the activation of T lymphocytes while simultaneously reducing the frequency of Tregs. The immune response that is induced by different dosages and fractions should be further explored to provide clinicians with information that would be valuable when making decisions regarding patient care.

Regarding cancer treatments, we have entered an era in which it is possible to achieve individualized comprehensive treatment. Successfully combining HSRT with other therapeutic regimens, including chemotherapy and immunotherapy, results in synergistic antitumor effects. It was previously demonstrated that the immune response and tumor reducing effects that are initiated by ablative RT treatment are abrogated by adjuvant chemotherapy but markedly amplified by local immunotherapy^18, 39^. Our results show that HSRT activates immune responses by enhancing the frequency of total T cells, especially the proportion of CD8^+^ T cells, and by inducing CD8^+^ T cells and CD4^+^ T cells to be reprogrammed into more functional cells by 3 weeks post-treatment. Moreover, the percentage of TNF-α^+^CD8^+^ T cells and IFN-γ^+^CD8^+^ T cells was not significantly altered after surgery (Fig. S3). In clinical practice, patients are routinely treated with adjuvant chemotherapy at 2 or 3 weeks post-RT. It is widely accepted that chemotherapy seriously inhibits patient immune functions, which could explain the poor results that have been observed when adjuvant chemotherapy is applied after HSRT. Indeed, it has been reported that when chemotherapy is provided as an adjuvant therapy after treatment with localized RT, it significantly hinders tumor regression and promotes melanoma outgrowth^40^.

It has been proposed that immunotherapeutic treatments should be administered after RT because RT results in the generation of *de novo* tumor antigens and breaks pre-existing peripheral immune tolerance^37^. However, administering RT after immunotherapy does offer certain advantages, including the activation of APCs and effector T cells and increased therapeutic effects^41^. Our data demonstrate that the immune response is more activated at 3 weeks after HSRT than immediately following the completion of HSRT, suggesting that the immune response that is induced by HSRT is dynamic and differs at different timepoints post-HSRT. Increasing our understanding of the immunologic effects that occur at different stages after HSRT would be useful for designing effecting combination therapies.

After HSRT, the percentages of B cells that were virgin, naïve B cells and double-negative B cells were lower, while the percentages that were MZ-like B cells, transitional B cells and plasmablast cells were higher. It was also reported that B cells with immunoregulatory functions were identified in both naïve and transitional B cell compartments and that T cell proliferation and effector function were suppressed in GVHD after cord blood transplantation^42^. However, the functions of B cells during anti-cancer immunity remain controversial. Determining the role of the changes that were observed in B cell subset compositions following HSRT will require further exploration.

In our study, we could recruit only older patients because the therapy guidelines recommend surgery for patients with stage I NSCLC. Immune parameters have been reported to decline with age^39^. In elderly lung cancer patients who were treated phytohemagglutinin (PHA), significant decreases were observed in the percentages of cells that were positive for CD3 or CD4, in the CD4/CD8 ratio and in lymphoblastogenesis (LB)^40^. However, our results demonstrate that HSRT activates the peripheral immune response in older patients with NSCLC. In the future, we will try to recruit younger patients to determine whether HSRT induces similar activating effects in the immune system.

Our results demonstrate that HSRT not only activates the immune response but also results in dynamic changes in the proportions of peripheral lymphocytes, which provides a clear benefit when determining the effectiveness of therapeutic regimens. First, our findings may explain why the OS for patients with operable stage I NSCLC was much higher for HSRT-treated patients than those treated with surgery. Our data indicate that HSRT is a better clinical option for stage I patients. Second, we demonstrate that an HSRT regimen of 48 Gy/6 F or 48 Gy/8 F induces immune system activation. Third, our results show that HSRT strongly activates the immune response at 3 weeks post-treatment. This may therefore be the ideal time at which to combine HSRT with other treatments, including chemotherapy and immunotherapy.

## Electronic supplementary material

Supplementary information.
