# Supplementary material for: Hypofractionated stereotactic radiation therapy activates the peripheral immune response in operable stage I non-small-cell lung cancer
Source: Sci Rep. 2017 Jul 7;7:4866. doi: 10.1038/s41598-017-04978-x (PMC5501824; doi:10.1038/s41598-017-04978-x)
Supplement: Supplementary file 1 — Supplementary information. [file 41598_2017_4978_MOESM1_ESM.doc]

**Supplementary Information**

**Hypofractionated stereotactic radiation therapy activates the peripheral immune response in operable stage I non-small-cell lung cancer**

Ting Zhang^1,4,y^, Haifeng Yu^2,y^, Chao Ni^3^, Tao Zhang^4^ , Luying Liu^4^, Qinghua Lv^4^, Zhigang Zhang^4^, Zhen Wang^4^, Dang Wu^1,4^, Pin Wu^4^, Guodi Chen^1^, Liancong Wang^1^, Qichun Wei^1^, Jian Huang^4^* and Xiaojian Wang^5^*

1. Department of Radiation Oncology, The Second Affiliated Hospital, Zhejiang University School of Medicine, Zhejiang University, Hangzhou 310009, China;

2. Department of Chemotherapy Center, Zhejiang Cancer Hospital, Hangzhou 310022, China;

3. Department of General surgery, Zhejiang Provincial People’s Hospital, Hangzhou 310014, China;

4. Cancer Institute (Key Laboratory of Cancer Prevention & Intervention, National Ministry of Education; Provincial Key Laboratory of Molecular Biology in Medical Sciences), Second Affiliated Hospital, Zhejiang University School of Medicine, Zhejiang University, Hangzhou 310009, China;

5. Institute of Immunology, School of Medicine, Zhejiang University, Hangzhou 310058, China.

**Supplemental Figure and legend:**


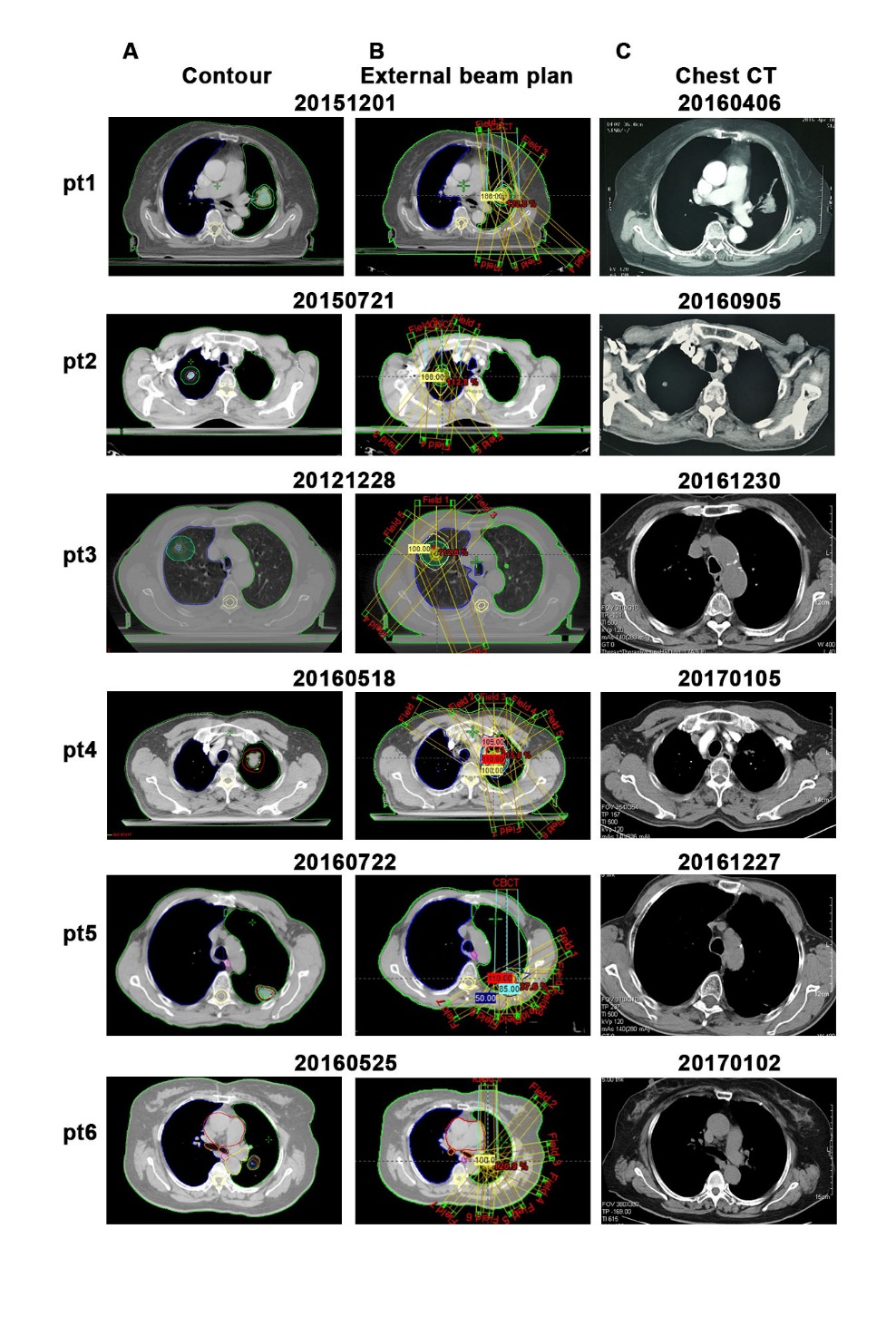


**Supplemental Figure 1. HSRT execution at the indicated time and the chest CT image at recent follow up**

Panel A: The contouring of GTV and CTV.

Panel B: Treatment planning of external beam radiotherapy for lung cancer.

Panel C: Chest computed tomography (CT) image showed the alteration of tumor after radiation therapy at the time of recent follow-up.

Pt:patient; GTV: gross tumor volume; CTV: clinical target volume.

**
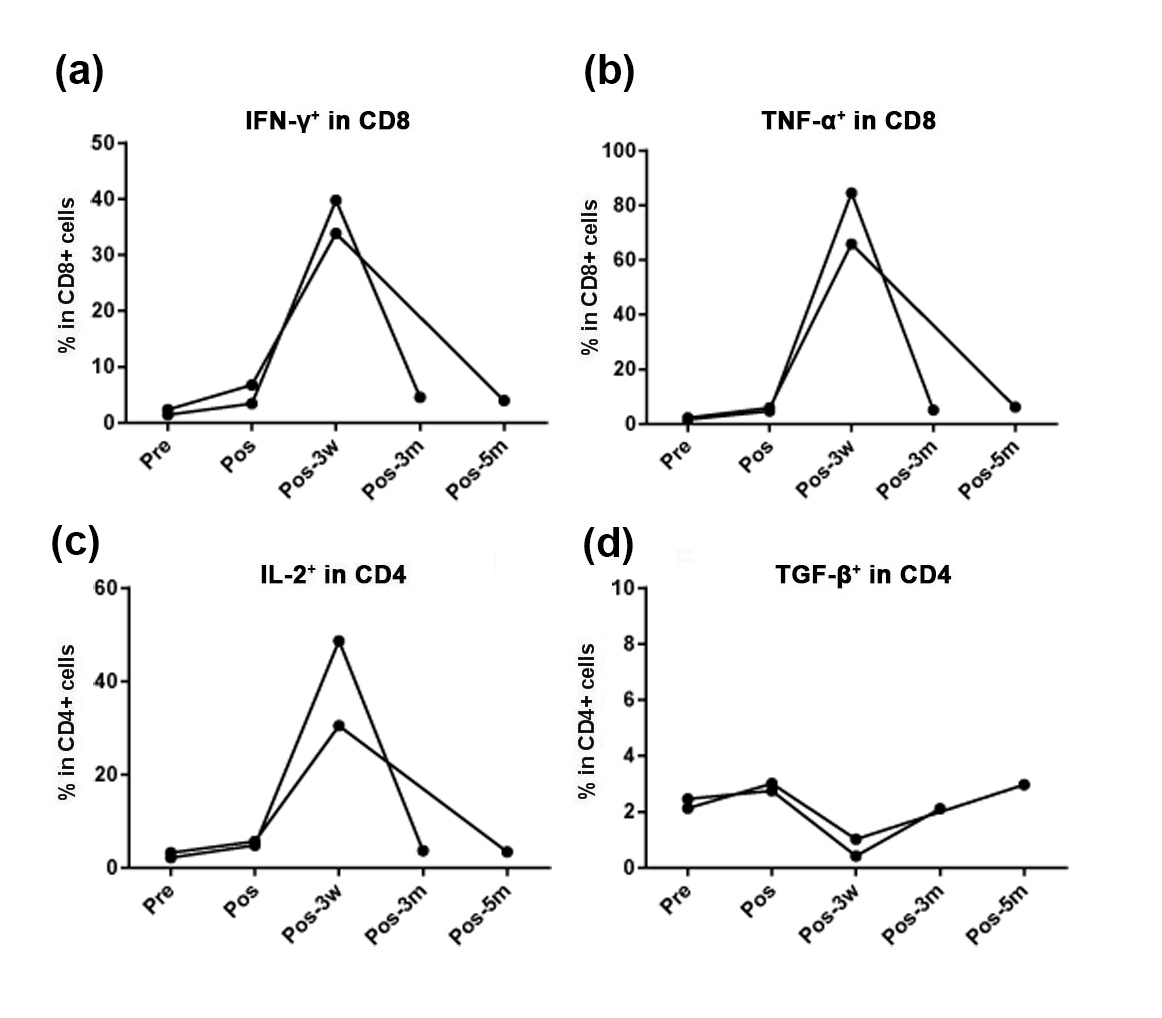
**

**Supplemental Figure 2. Cytokine secretion by peripheral T cells after treatment with HSRT.** PBMCs were collected from two recruited patients at the indicated times and were treated with a leukocyte activation cocktail for 6 h, and the cells were then collected and labeled with IFNγ, TNFα, IL-2, TGFβ. The frequencies of IFNγ^+^CD8^+^ T cells in CD8^+^ T cells (a), TNFα^+^CD8^+^ T cells in CD8^+^ T cells (b), IL-2^+^CD4^+^ T cells in CD4^+^ T cells (c), TGFβ^+^CD4^+^ T cells in CD4^+^ T cells (d) were analyzed at the indicated times.

**
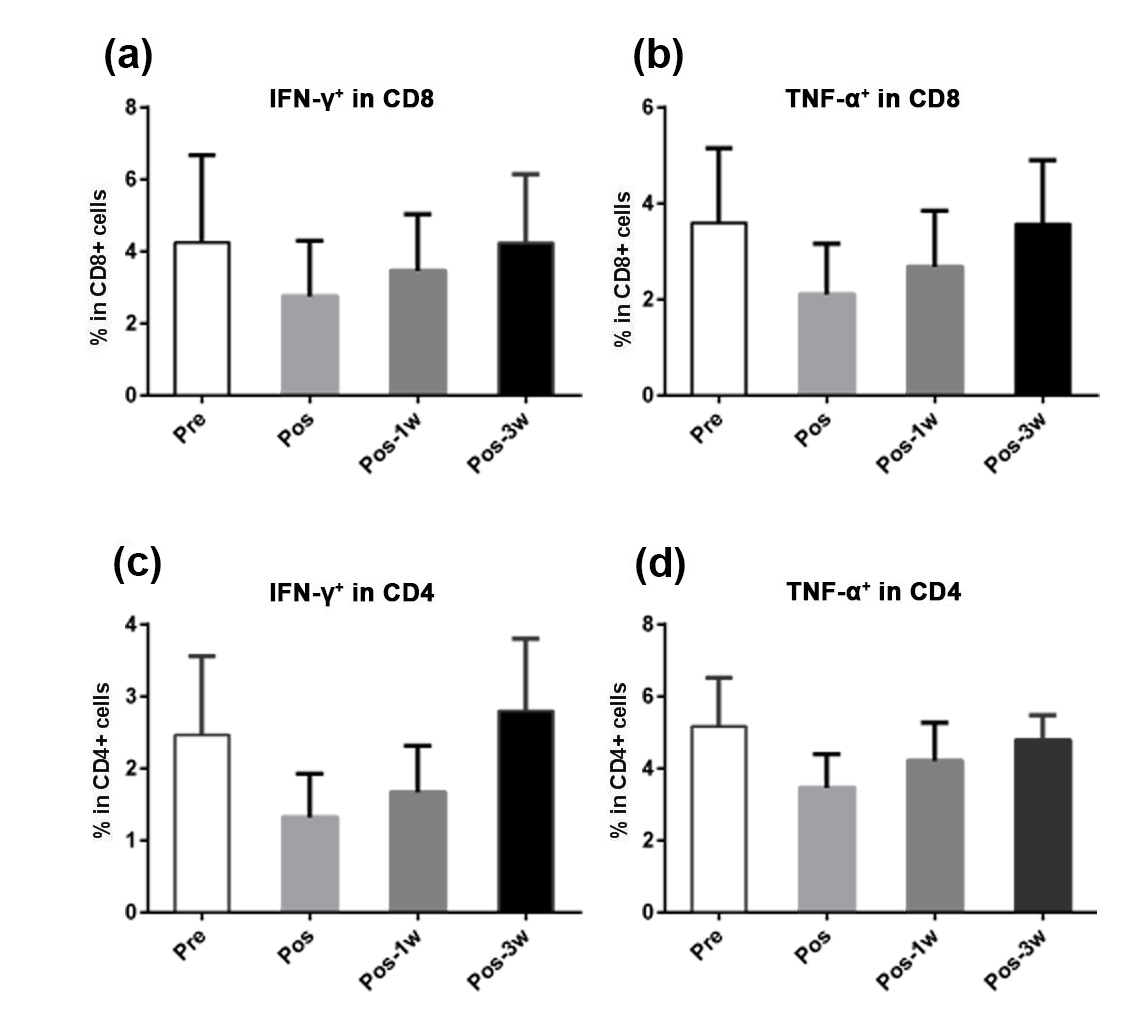
**

**Supplemental Figure 3. Cytokine secretion by peripheral CD8^+^ T cells after treatment with surgery.** Peripheral blood samples were obtained from the 5 NSCLC patients on the day before surgery (pretreatment, pre), the second day after surgery (post-treatment, pos), at 1 week post-surgery (pos-1w) and 3 week post-surgery (pos-3w). PBMCs were collected and were treated with a leukocyte activation cocktail for 6 h, and the cells were then collected and labeled with IFNγ, TNFα. The frequencies of IFNγ^+^CD8^+^ T cells in CD8^+^ T cells (a), TNFα^+^CD8^+^ T cells in CD8^+^ T cells (b), IFNγ^+^CD4^+^ T cells in CD4^+^ T cells (c),TNFα^+^CD4^+^ T cells in CD4^+^ T cells (d) were analyzed at the indicated times.

**Supplemental Table 1: Patients’ follow up data**

| Patients | Radiotherapy | Recent follow-up | | |
| --- | --- | --- | --- | --- |
|  |  | Time | Inspection item | Results |
| Pt1 | 20151201 | 20160416 | Chest CT  Brain MRI  [Abdominal ultrasound](http://www.baidu.com/link?url=bMwvk4wFkc7lm3k5FgMGo1DAYv0l2emS-BN_RIiEcnTD4bh-oGf_TlcLOaVosUkMkWLPj9sj7E_D6CSSJEhyEb9WrIn169m-v48H7HXch8y5Bt1ejZpghXz82WBXVyFc) | No progression |
| Pt2 | 20150721 | 20160905 | Chest CT  Brain MRI  [Abdominal ultrasound](http://www.baidu.com/link?url=bMwvk4wFkc7lm3k5FgMGo1DAYv0l2emS-BN_RIiEcnTD4bh-oGf_TlcLOaVosUkMkWLPj9sj7E_D6CSSJEhyEb9WrIn169m-v48H7HXch8y5Bt1ejZpghXz82WBXVyFc) | No progression |
| Pt3 | 20121228 | 20161230 | Chest CT  Liver MRI | No progression |
| Pt4 | 20160518 | 20170105 | Chest CT  [Abdominal ultrasound](http://www.baidu.com/link?url=bMwvk4wFkc7lm3k5FgMGo1DAYv0l2emS-BN_RIiEcnTD4bh-oGf_TlcLOaVosUkMkWLPj9sj7E_D6CSSJEhyEb9WrIn169m-v48H7HXch8y5Bt1ejZpghXz82WBXVyFc) | No progression |
| Pt5 | 20160722 | 20161227 | Chest CT  [Abdominal ultrasound](http://www.baidu.com/link?url=bMwvk4wFkc7lm3k5FgMGo1DAYv0l2emS-BN_RIiEcnTD4bh-oGf_TlcLOaVosUkMkWLPj9sj7E_D6CSSJEhyEb9WrIn169m-v48H7HXch8y5Bt1ejZpghXz82WBXVyFc) | No progression |
| Pt6 | 20160525 | 20170102 | Chenst CT  [Abdominal ultrasound](http://www.baidu.com/link?url=bMwvk4wFkc7lm3k5FgMGo1DAYv0l2emS-BN_RIiEcnTD4bh-oGf_TlcLOaVosUkMkWLPj9sj7E_D6CSSJEhyEb9WrIn169m-v48H7HXch8y5Bt1ejZpghXz82WBXVyFc) | No progression |

**CT: Computed Tomography; MRI: Magnetic Resonance Imaging**

**Supplemental Table2:**

**Antibodies for Flow Cytometry**

| Reagents | Clone | Manufacturers |
| --- | --- | --- |
| anti-CD3-PECy7 | UCHT1 | BioLegend |
| anti-CD3-PerCP-Cy5.5 | HIT3a | BioLegend |
| anti-CD4-PerCP-Cy5.5 | A161A1 | BioLegend |
| anti-CD8-APCCy7 | HIT8a | BioLegend |
| anti-Vδ1 TCR-FITC | TS8.2 | GeneTex |
| anti-Vδ2 TCR-PerCP-Cy5.5 | B6 | BioLegend |
| anti-CD16-Alexa Fluor 647 | 3G8 | BioLegend |
| anti-CD25 (IL-Rα)-PE | BC96 | BioLegend |
| anti-CD45-APCCy7 | HI30 | BioLegend |
| anti-CD56-PECy7 | HCD56 | BioLegend |
| anti-CD14-PerCp-Cy5.5 | 63D3 | BioLegend |
| anti-BDCA-2-PE | 201A | BioLegend |
| anti-CD11c-PECy7 | 3.9 | BioLegend |
| anti-CD20-FITC | 1412 | BioLegend |
| anti-CD20-PECy7 | 1412 | BioLegend |
| anti-IgG-APC | HP6017 | BioLegend |
| anti-IgM-FITC | MHM-88 | BioLegend |
| anti-IgD-PE | IA6-2 | BioLegend |
| anti-CD5-PE | L17F12 | BioLegend |
| anti-CD21-APC | Bu32 | BioLegend |
| anti-CD22-FITC | HIB22 | BioLegend |
| anti-CD23-PECy7 | EBVCS-5 | BioLegend |
| anti-CD27-PerCP-Cy5.5 | LG.3A10 | BioLegend |
| anti-CD24-APC | ML5 | BioLegend |
| anti-CD38-PECy7 | HB-7 | BioLegend |
| anti-TNF-α-APC | MAb11 | BioLegend |
| anti-IFN-γ- Alexa Fluor 488 | 4S.B3 | BioLegend |
| anti-TGF-β-FITC | TW4-2F8 | BioLegend |
| anti-IL-9-PE | MH9A4 | BioLegend |
| anti-IL-1β-Alexa Fluor 647 | H1b-98 | BioLegend |
| anti-IL-2-APC | MQ1-17H12 | BioLegend |
| anti-IL-22-PE | 2G12A41 | BioLegend |
| anti-Foxp3-PECy7 | 206D | BioLegend |
| anti-GranzymB-FITC | GB11 | BioLegend |
